# Views of healthcare professionals on the inclusion of genes associated with non-syndromic hearing loss in reproductive genetic carrier screening

**DOI:** 10.1038/s41431-022-01239-y

**Published:** 2023-02-09

**Authors:** Lucinda Freeman, Martin B. Delatycki, Jackie Leach Scully, Nancy Briggs, Edwin P. Kirk

**Affiliations:** 1 School of Women’s and Children’s Health, UNSW, Randwick, NSW Australia; 2 Graduate School of Health, University of Technology Sydney, Sydney, NSW Australia; 3 Murdoch Children’s Research Institute, Parkville, VIC Australia; 4 Victorian Clinical Genetics Services, Parkville, VIC Australia; 5 Disability Innovation Institute, UNSW, Randwick, NSW Australia; 6 Stats Central, Mark Wainwright Analytical Centre, UNSW, Randwick, NSW Australia; 7 Centre for Clinical Genetics, Sydney Children’s Hospitals Network NSW, Sydney, NSW Australia; 8 NSW Health Pathology East Genomics, Randwick, NSW Australia

**Keywords:** Genetic testing, Ethics

## Abstract

Genes associated with non-syndromic hearing loss (NSHL) are frequently included in panels for reproductive genetic carrier screening (RGCS), despite a lack of consensus on whether NSHL is a condition appropriate for inclusion in RGCS. We conducted a national online survey using a questionnaire to explore the views of clinicians who facilitate RGCS or provide care to deaf individuals in Australia and New Zealand regarding the inclusion of such genes in RGCS. Results were analysed descriptively, and free-text responses were analysed thematically. The questionnaire was completed by 386 respondents including genetic healthcare providers, obstetricians, ear nose and throat specialists, and general practitioners. The majority of respondents agreed that genes associated with NSHL should be included in RGCS, but there were differences between the groups. 74% of clinicians working in a hearing clinic agreed these genes should be included compared to 67% of genetic healthcare providers, 54% of reproductive care healthcare providers, and 44% of general practitioners. A majority of respondents agreed that moderate to profound deafness is a serious disability, although genetic healthcare providers were less likely to agree than other groups. Overall, respondents agreed that including NSHL in RGCS upholds prospective parents’ right to information. However, they also identified major challenges, including concern that screening may express a discriminatory attitude towards those living with deafness. They also identified the complexity of defining the severity of deafness.

## Introduction

Advances in testing technology have made it possible to include large panels of genes in reproductive genetic carrier screening (RGCS). Deciding which genes to include in RGCS is a complex issue, but there is general acceptance that the intent is not to identify prospective parents who have an increased chance of having a child with a mild condition. There is general agreement in the literature that the intent is to identify pregnancies at risk of severe conditions, and conditions should only be included in a RGCS panel if there is a well-defined phenotype with onset early in life, and if criteria of severity are met [1–4]. Whilst there are no professional guidelines on which conditions to include in screening, several groups have considered conditions appropriate for inclusion if they meet some or all of a list of clinical criteria including the presence of shortened life expectancy, cognitive impairment, physical disabilities, or a need for burdensome treatment [5, 6]. Screening for such conditions enables prospective parents to make informed reproductive decisions to avoid or prepare for the birth of a child with the condition.

The inclusion of mild and/or variable conditions in such screening is contentious, in part because this information may not be as useful for reproductive decision-making [7–9]. There is no consensus definition of disease severity and groups have made different judgements about which conditions are severe enough to include in screening. At a societal level, offering screening for a genetic condition may send a message that using reproductive options to avoid the condition is appropriate [10]. Programs that screen for a large number of conditions, not all considered severe, have been criticised as they can be perceived to express a discriminatory attitude towards those who live with the condition [11, 12].

One condition whose inclusion has been controversial is non-syndromic hearing loss (NSHL) [1]. It is noted that 18/23 commercial RGCS panels include *GJB2*, the gene most commonly associated with autosomal recessive NSHL [1]. Despite the inclusion of *GJB2* on many RGCS expanded panels, attitudes towards screening for NSHL in the general ‘low-risk’ population remain unclear. Data on the reproductive choices made by couples at increased chance of having a child who would be deaf are limited, but it has been reported that some have used preimplantation genetic testing or prenatal diagnosis to avoid having a child who is deaf, whilst others have not altered their reproductive plans [13].

Hearing loss is the most common neurosensory deficit in children in developed countries and creates challenges to development and quality of life for affected individuals [14]. Early diagnosis and intervention, however, have been shown to reduce developmental challenges and enhance quality of life for children who are born deaf [15]. For this reason, newborn hearing screening has been widely implemented and proven successful at identifying babies with moderate-to-severe hearing loss [16]. Autosomal recessive causes of NSHL are common, with approximately 1 in 50 individuals being carriers of a pathogenic variant of *GJB2*, meeting the frequency criterion for RGCS in the recent American College of Medical Genetics practice guideline [5].

As RGCS is potentially relevant to anyone planning a pregnancy or in early pregnancy, most people accessing RGCS will not have lived experience of a condition. There is a reliance on, and trust in, the decision-makers who choose which conditions are included in RGCS panels as consumers will expect that any information gained from screening will be useful to them [17]. The broader implications of the inclusion of each condition need careful consideration and consultation. Although public policy on RGCS should not be based solely on the clinical perspectives, it is prudent to consider the views of those who will be discussing RGCS with prospective parents and of clinicians who provide care for deaf children.

Despite its clear relevance, there is currently little knowledge of the attitudes of healthcare professionals (HCP) towards RGCS for NSHL. This study sought to understand the views of HCP on the inclusion of NSHL in RGCS and their views on the severity of NSHL as a health condition. The study looked at NSHL as an exemplar of the many moderate or mild health conditions that may be considered for inclusion in RGCS.

## Methods

### Survey development

The survey was developed to explore the views of HCP on inclusion of genes for NSHL in RGCS and was exploratory rather than hypothesis driven (S1). In addition to demographic questions and knowledge questions, eight agree-disagree statements were designed to assess the key issues in the inclusion of genes associated with NSHL in a population-wide RGCS program. A consumer representative and several HCP providing reproductive care and care for those with hearing loss were consulted in preparing the questionnaire. The survey collected data on (1) knowledge and experience of NSHL and RGCS; (2) attitudes to inclusion of NSHL in RGCS; and (3) views on the impact of deafness on a child. The survey took approximately 10 min to complete and was approved by the Sydney Children’s Hospital Network Human Research Ethics Committee, pilot tested with three genetic counsellors and distributed (between November 2020 and October 2021) via HCP organisations.

### Survey recruitment

HCP were recruited using two approaches:

i. Genetic HCP (genetic counsellors and clinical geneticists) were recruited through the Australasian Society of Genetic Counsellors and the Human Genetics Society of Australasia; obstetricians and gynaecologists (including fertility specialists) through the Royal Australian and New Zealand College of Obstetricians and Gynaecologists; HCP working in hearing clinics (including ear nose and throat specialists and paediatricians) through the Childhood Hearing Australasian Medical Professionals Network and the Australasian Newborn Hearing Committee; and general practitioners through several webcasts organised by HealthEd, a private medical education company that organises seminars and learning resources for GPs. The webcasts covered topics unrelated to NSHL or RGCS.
ii. Dissemination through directors of hearing support clinics across Australia to snowball the invitation to professionals in their network likely to work with deaf children.

Governing bodies did not permit direct recruitment from organisation membership. As a result, the number of HCP in each group who received the invitation (i.e., the denominator) is unknown, however approximate numbers of invitations can be estimated from the professional organisational membership numbers at the time of the study. These were reported as 1276 for the Human Genetics Society Australasia; 5000 for the Royal Australian and New Zealand College of Obstetricians and Gynaecologists; and 43 for the Childhood Hearing Australasian Medical Professional Network. HeathEd webinars report average attendance of between 100 and 200 General Practitioners at each webinar.

### Statistics and data analysis

Survey data were collected, stored, and managed in Research Electronic Data Capture Version 10.0.1 [18] hosted at the University of New South Wales. Descriptive statistics were computed for all items. Respondents were grouped for analysis using medical field of practice (genetic HCP; reproductive care HCP; general practitioner; and hearing support HCP). Statistical analysis was performed using IBM Statistical Package for the Social Sciences software (SPSS version 23.0, SPSS Inc., Chicago, IL) and R, version 4.1.2 [19]. Categorical data were reported as frequencies and percentages with pairwise differences from an ordinal logistic regression were calculated. *P*-values for pairwise comparisons in the regressions were adjusted for multiple comparisons using Tukey’s method. Statistical significance was assessed at *p* < 0.05.

Respondents were asked one open-ended question on the topic, and the free text answers were separated according to professional group in Excel (Microsoft). Thematic analysis [20] was used to interpret the free text comments and identify themes relating to the inclusion of NSHL in RGCS. Coding and analysis were checked by LF, MD, and EK until consensus was reached.

## Results

A total of 386 health professionals completed the questionnaire. Participant information was provided at the start of the survey, and informed consent was implied by voluntary completion of the survey. The majority of participants identified as female (*N* = 271, 69%). Across all professions, 14% (*N* = 54) had worked less than five years, 45% (*N* = 174) had worked between six and twenty years, and 41% (*N* = 174) had worked in their profession for over 20 years. HCP were grouped into fields of practice to allow for comparison of professions. These were (i) Genetic HCP (*N* = 94; Genetic counsellors and clinical geneticists); (ii) Reproductive care HCP (*N* = 153; Obstetricians, gynaecologists and fertility specialists); (iii) General practitioners (*N* = 103); and (iv) Hearing support staff (*N* = 34; audiologists, ENT specialists, neonatologists, paediatricians and newborn hearing coordinators) (see Table 1).

**Table 1:** Demographics of respondents.

Demographic	*N*	%
Sex		
Female	267	69%
Male	113	29%
Prefer not to say	6	2%
**Years working in profession**		
0–5 y	54	14%
6–10 y	72	19%
11–20 y	102	26%
20 + y	158	41%
**Healthcare Profession**		
**General Practitioner**	103	27%
**Genetics**		
Genetic Counsellors	74	19%
Clinical Geneticists	20	5%
**Reproductive Care**		
Obstetrician	122	32%
Obstetrician Registrar	11	3%
Fertility specialist	20	5%
**Hearing clinic**		
Audiologist	8	2%
Neonatologist (running hearing clinic)	2	0.5%
Newborn hearing screening coordinator	1	0.3%
Paediatrician	17	4%
Ear Nose Throat Specialist	6	2%
Unknown	2	0.5%

The majority of respondents, *N* = 319 (83%), have ordered RGCS for their patients and the majority (*N* = 303, 79%) intend to order RGCS for patients in the future.

### Perceptions of childhood bilateral moderate-to-profound deafness

**Fig. 1:**
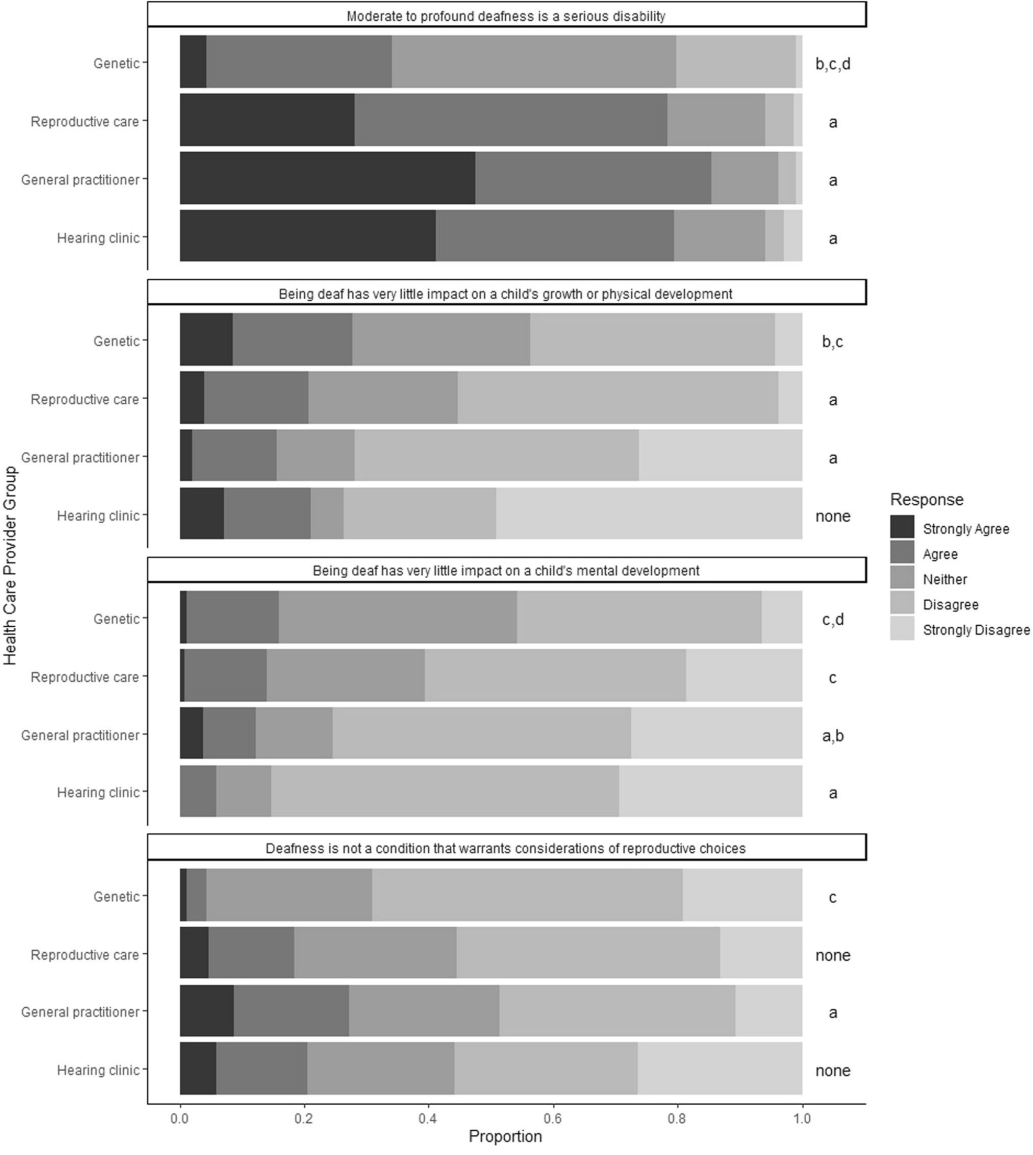
Attitudes of different HCP groups towards the severity of deafness and reproductive choices for deafness. Views regarding the severity of deafness and reproductive choices for deafness are presented stratified by healthcare provider groups. Proportions of responses on the likert scale (strongly agree/agree/neither agree nor disagree/ agree/strongly agree) are represented. Significant differences (*p* < 0.05) are noted in the plot, with the letters indicating group differences. Letters indicate a significant difference versus: **a** Genetic Healthcare providers; **b** Reproductive Healthcare providers; **c** General Practitioners, and **d** Hearing clinic healthcare providers.

Overall the majority of respondents (69%, *n* = 296) agreed that moderate-to-profound deafness is a serious disability, while only 9% (*n* = 34) of respondents disagreed with this statement (see Fig. 1). Nevertheless, attitudes on whether moderate-to-profound deafness is a serious disability were significantly different between the groups of HCP (*p* < 0.05), with the genetic HCP overall less likely to agree that deafness is a serious disability.

Although a majority in all HCP groups disagreed with the statement that being deaf has little impact on a child’s physical and mental development, there was a significant difference between the HCP groups (*p* < 0.05). The genetic HCP and the hearing clinic HCP were more likely than the other groups to agree that deafness has little impact on a child’s growth and development. Both genetic HCP and hearing clinic HCP agree that deafness has an impact on mental development, and the hearing clinic HCP report this more than the genetic HCP. Across all HCP, 21% of respondents (*N* = 82) agreed that deafness has very little impact on a child’s growth or physical development but only 13% (*N* = 52) agreed that it has very little impact on a child’s mental development.

Responses to the statement about deafness being a disadvantage rather than a disability were more divided, with 37% (*N* = 140) of participants agreeing that it is a ‘disadvantage but not a disability’ while 43% (*N* = 160) disagreed with this statement. The majority across all HCP groups (77%, *N* = 296) agreed that there are good treatment and management options for children who are deaf, with only a very small number (4%, *N* = 14) disagreeing with this statement.

After controlling for profession, when attitudes of respondents were analysed in a multiple logistic regression model, disagreement that NSHL is a serious disability was associated with lower odds of agreement that genes associated with NSHL should be included in RGCS ((*p* < 0.0001); Neither OR = 0.35 (CI: .21–.60); Disagree OR = 0.19 (CI = 0.08–1.43)) and higher odds of agreement that deafness is not a condition that warrants consideration of reproductive choices (*p* = 0.001; Neither OR = 2.84 (CI: 1.62–5.34); Disagree OR = 2.05 (CI = .79–5.34)). Controlling for profession, there was no association between views on the impact of deafness on a child’s mental wellbeing (*p* = .182) or on their physical growth and development (*p* = .369) and the response to the inclusion of deafness in RGCS.

### Attitudes to including non-syndromic hearing loss in RGCS

**Fig. 2:**
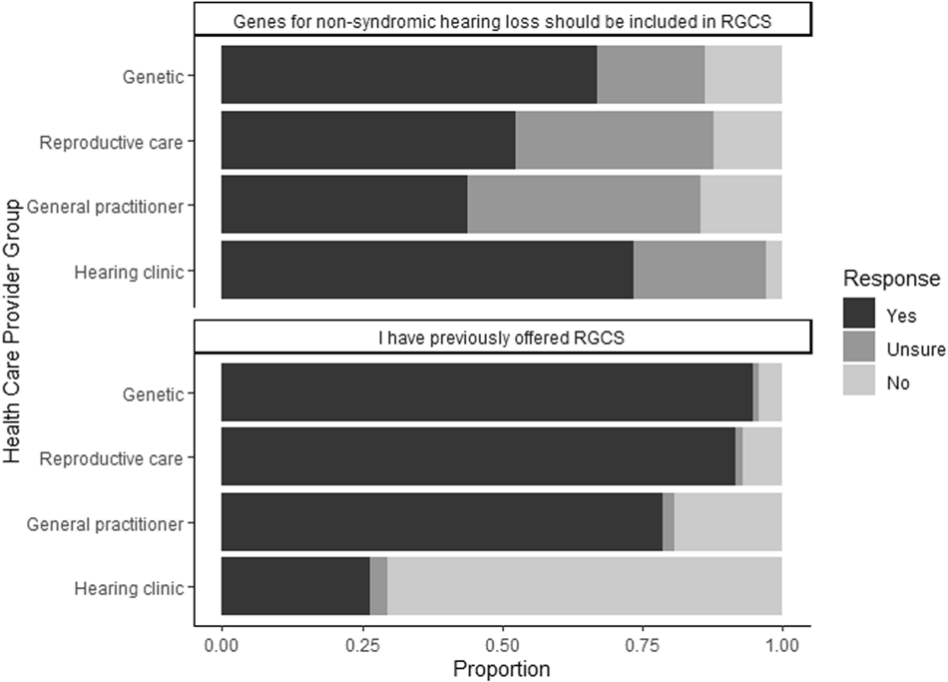
Attitudes of different healthcare provider groups towards the inclusion of genes associated with non-syndromic hearing loss in reproductive genetic carrier screening. Values are presented as proportions of the total responses.

Overall, a small majority of all respondents (55%, *N* = 213) agree that deafness should be included in RGCS while the remainder are mostly unsure (32%, *N* = 125) rather than unsupportive (12%, *N* = 48) (see Fig. 2). There was a significant difference (*p* = 0.004) between the groups on the issue of whether genes for NSHL should be included in RGCS. The hearing clinic HCP (67%; *N* = 25) and genetic HCP (67%; *N* = 63) were more likely to agree that they should be included than reproductive care HCP (52%; *N* = 80), or general practitioners (44%; *N* = 45). However, a very small proportion of hearing clinic HCP (3%) and less than 15% of the other HCP groups disagree with this statement; i.e., they do not support inclusion of genes for NSHL in RGCS

Most participants (79%, *N* = 304) agree that couples in the general population would want to know about their chances of having a deaf child. Similarly, most respondents (86%; *N* = 331) agree that couples should be able to choose whether to learn about their chances of having a deaf child.

The majority (57%; *N* = 220) disagree with the statement that deafness is not a condition that warrants consideration of reproductive choices.

However, there was also a significant difference when comparing across all HCP groups (*p* = 0.016). Genetic HCP were much more likely to disagree with this statement, i.e., to support the idea that deafness is a condition that warrants consideration of reproductive choices.

Analysis shows no significant correlations between the responses and how much exposure a clinician has had to providing care for a deaf patient in the past.

### Thematic analysis

Although the quantitative responses indicated strong support for including NSHL in RGCS, there was considerable nuance in the free text comments, highlighting implementation challenges that are both principled and practical in nature. Free text responses were received from 122 HCP (32%). These were analysed for common patterns and further abstracted into several themes representing both concerns about and benefits of the inclusion of genes associated with NSHL in RGCS.

Five major themes emerged from the free text comments in this survey: (1) Parental choice is facilitated through informed decision making; (2) inclusion of NSHL in a RGCS panel is akin to eugenics and also expresses a discriminatory attitude towards those living with deafness; (3) defining the severity of deafness is complex; [21] need for wider consultation on this topic with people who have a lived experience of deafness; [5] there should be consideration of limitations on the reproductive decisions available to prospective parents after screening for NSHL. A selection of quotes is given to illustrate these themes (further quotes can be found in Supplementary material S1). Whilst the complexity of defining the severity of deafness was a theme represented across all HCP groups, the other themes were identified within specific HCP groups as seen in Box 1.

#### Parental choice is facilitated through informed decision making

Most of the genetic HCP advocated for inclusion of genes associated with NSHL into a wider RGCS panel based on giving prospective parents the information to make their own informed choice. However, some respondents were uncertain how NSHL-linked genes could be included as an optional test, even with a separate process of consent. Genetic HCP were more likely to comment on the need to provide information and choice to prospective parents.

*I strongly believe in a couple’s right to informed decision making - both regarding choosing whether to include deafness on their carrier screening test, and their right to reproductive decision making on the basis of that information.* *Genetic Counsellor*

#### Inclusion of NSHL in a RGCS panel is akin to eugenics and can also express a discriminatory attitude to those living with deafness

GPs and reproductive care HCP were more likely to raise concerns that align with both the eugenics critique and the ‘expressivist’ critique of prenatal selection. Comments highlighted a concern that including NSHL in RGCS would express a discriminatory attitude towards those living with deafness, and that prospective parents should not be able to select the type of people who will be born. This tension was further expanded on by respondents who believe that deafness is not a condition for which termination of pregnancy is warranted.

*Population-based carrier screening for any condition, whether associated with mild to severe disability, casts the condition and persons affected by the condition in a particular, usually negative, light. further [it] is highly discriminatory and derogatory towards those persons affected by those conditions.* *GP*

Genetic HCP were more likely to comment on the potential for routinisation; if NSHL is included in screening, prospective parents may feel obliged to follow a path of further testing.

*I worry that prospective parents will feel pressure to use prenatal testing or PGD to exclude deafness because it is included in carrier screening tests.* *Genetic Counsellor*

#### Defining the severity of deafness is complex

Many respondents raised the difficulty of defining how severe deafness is as health condition or disability. Whilst they acknowledged the multitude of factors involved (socio-environmental context, cultural differences) they also noted that the perception of severity is based on personal experiences and that this can vary greatly.

*… it is difficult to make generalised statements about the quality of life of a deaf child / person.* *Obstetrician*

#### There is a need for wider consultation on this topic with people who have a lived experience of deafness

Several respondents acknowledged that the views of HCP are not necessarily the most important voice in this discussion and that there is a need for wider consultation with people who have lived experience of deafness.

*I don’t feel like I am the right person to say whether or not it should be included. Perhaps asking the Deaf community…* *Genetic Counsellor*

#### There should be limitations on the reproductive decisions available to prospective parents if NSHL is included in RGCS

Some respondents feel that if screening for NSHL is possible, the information should be available to facilitate planning and preparing for the birth of a deaf child but not for any decisions aiming to avoid having a deaf child. Others suggested that the information should be restricted to use in preimplantation genetic testing with IVF but not in prenatal testing and termination of pregnancy.

*I have some concerns about deafness being a sufficient reason to terminate a pregnancy… Deafness doesn’t shorten a lifespan or cause suffering. I don’t think terminating a potentially deaf baby is appropriate.* *General practitioner*

Box 1 Themes identified through thematic analysis of free text comments across the different healthcare (HCP) provider groups

1. **Genetic HCP**

- Defining the severity of deafness is complex.
- Parental choice is facilitated through informed decision-making.
- Need for wider consultation with people who have a lived experience of deafness.

2. **Reproductive care HCP**

- Defining the severity of deafness is complex.
- Including deafness in RGCS expresses a discriminatory attitude to those living with deafness.
- Limitations needed on reproductive decisions available for NSHL.

3. **General practitioners**

- Defining the severity of deafness is complex.
- Including deafness in RGCS expresses a discriminatory attitude to those living with deafness.
- Limitations needed on reproductive decisions available for NSHL.

4. **Hearing clinic HCP**

- Defining the severity of deafness is complex.
- Parental choice is facilitated through informed decision-making.

## Discussion

This cross-sectional survey provides important insights into the knowledge of and attitudes towards the inclusion of genes associated with NSHL in RGCS among genetic clinicians, general practitioners, reproductive care specialists, and hearing clinic HCP. When attitudes of respondents were analysed in a multiple logistic regression model, a greater level of disagreement with the idea of deafness as a serious disability was significantly associated with less support for inclusion of genes associated with NSHL in RGCS, and with the statement that deafness is not a condition that warrants consideration of reproductive choices. Whilst a majority of all clinicians responding to the survey agree that genes associated with NSHL should be included in RGCS, there were significant concerns expressed about the availability of reproductive options for avoiding the birth of children with deafness and the possibility that inclusion of NSHL-linked genes would express a discriminatory attitude to those living with deafness.

Our sample is unique because it captures HCP across different disciplines who may be involved in ordering RGCS, discussing test results, and/or managing children with NSHL. The greatest divergence of views was between HCP working in a hearing clinic and genetic HCP. Whilst all clinicians were familiar with deafness and had provided care on some level for patients with moderate to profound hearing loss, the genetic HCP were least likely to agree that it is a serious disability. Although both genetics HCP and HCP working in hearing clinics see children with severe conditions, the mix is likely to be different, with genetics HCP spending a greater proportion of their time seeing patients who are more seriously impacted by medical and cognitive challenges in addition to hearing loss. This may be a partial explanation for the difference between the professional groups. This result highlights the subjectivity of HCPs’ attitudes, that may be influenced by their comparative exposure to/experience of illness and disability. Despite the finding that genetic HCP do not perceive NSHL to be as severe a condition as other HCP groups, it may be the strong desire to promote autonomy and informed choice as revealed in the thematic analysis supports their inclination to include genes associated with NSHL in RGCS.

The views of HCP about the impact of NSHL on the physical and mental development of children varied widely. For example, the hearing clinic HCP were more likely than genetic HCP to perceive deafness as having a significant impact on the mental development of children. Thus, achieving any consensus for policymaking is likely to be difficult. In line with this, it has been demonstrated that genetics professionals do not always agree about the seriousness of genetic conditions [22, 23]. There is a spectrum of opinions rather than a clear division, and basing RGCS policies on clinicians’ views alone would be challenging and could complicate implementation of population-wide RGCS.

The questionnaire results demonstrate the difficulties of attempting to define the severity of NSHL. This is significant for RGCS as it is often genetic [1, 24] and reproductive care HCP [1, 5, 24] who have been involved in writing the recommendations and guidelines for which conditions to include in screening. As most people undergoing RGCS will not have a lived experience of deafness, they rely on the expertise of those who choose the conditions to be included in RGCS. Consumers will have an expectation that any information gained from screening will be useful to them. As demonstrated by our findings, there is no consensus on the perception of severity of moderate to profound deafness between different HCP groups. Deafness has previously been classified as a moderately severe condition [7, 9, 13] but was excluded from a recently published extended RGCS gene panel used in Australia, with some on the gene selection committee of the view that these genes should be included and others of the opposite view [1]. We found that whilst most of our respondents consider deafness to be a serious health condition, they also think that the available medical care means it need not have a significant impact on overall quality of life.

The importance of considering the perspective of those with a personal experience of deafness was also expressed here, but only by genetic HCP who, because of their training, recognise that deafness can be experienced differently by deaf individuals and their families and that these diverse perspectives should be taken into account when selecting conditions for RGCS. Empirical research reveals that people who live with genetic conditions and their families often have views on the severity of their condition that differ significantly from those not familiar with the lived experience of the condition [25]. Whilst clinician views have often been prioritised in selecting which genes to include in RGCS, there is a growing body of evidence recognising the importance of the patient’s perception of the seriousness of a condition [26].

Offering screening for a genetic condition sends an implicit message that there are valid reasons for avoiding the birth of a child with that condition, or that knowing about the condition prenatally can be beneficial for clinical management. Some respondents in this study were concerned that inclusion of genes associated with NSHL in RGCS expresses a discriminatory attitude towards those living with deafness. This is not the only study to question the potential for harm from the use of genetic technologies for deafness in the reproductive setting, threatening the future of the culturally Deaf community and the loss of a form of cultural diversity [27–29]. Studies of individuals who are deaf and of hearing people who have indirect experience of deafness (children of deaf adults and parents of deaf children) show that they may feel that genetic testing for deafness in the reproductive setting expresses a negative view of deafness [29–31].

Whilst some HCP expressed concerns about the inclusion of NSHL genes they also identified benefits, such as increasing parental choice through informed decision-making. Providing education about the screening test and the included conditions is paramount for individuals to be able to make an informed decision. As evidenced in this study, it may prove difficult to include deafness in a statement that tells prospective parents that RGCS will only screen for serious, childhood-onset conditions.

### Strengths and limitations

The study was strengthened by participants’ anonymity, increasing the likelihood of truthful responses. A potential weakness of our study is that the survey has not previously been validated and was exploratory in design. A response bias is possible if individuals with experience of either NSHL or RGCS were more likely to respond to the survey.

The provider population of this study may not be representative of the wider healthcare provider populations due to the relatively low number of respondents. Our participant group is skewed towards female practitioners which is not reflective of the broader provider demographics in Australia. The design of the study allowed for differences in attitudes between the various HCP groups to be observed but did not allow reasons for these differences to be determined.

## Conclusion

Our study provides a useful insight into the attitudes of different HCP in Australia towards the inclusion of NSHL in RGCS. It shows that there are mixed views on whether NSHL should be included in RGCS and, if it is included, whether it should be an optional screening test set aside from an expanded panel that focuses on conditions generally considered more severe.

This study highlights a need to be cautious if genes associated with NSHL are included on a testing panel without any separate consent process or provision of information specific to the lived experience of NSHL. Further consultation is needed from those with a lived experience of deafness to gain a deeper and more comprehensive insight into the benefits and harms of including NSHL in RGCS.

Many of the issues identified in this study are likely to be relevant both to other countries and to other conditions, particularly if a government-funded population wide approach to screening is in place or being considered. The authors are currently conducting further research with other stakeholder groups to explore the attitudes of the general public interested in RGCS and those with a lived experience of deafness towards carrier screening for NSHL, in order to further guide the discussion.

## Supplementary information

Supplementary Material 1

Supplementary Material 2

## Data Availability

Data analysed during this study are not published but may be available from the corresponding author on reasonable request.
