# Supplementary material for: Views of healthcare professionals on the inclusion of genes associated with non-syndromic hearing loss in reproductive genetic carrier screening
Source: Eur J Hum Genet. 2023 Feb 9;31(5):548–54. doi: 10.1038/s41431-022-01239-y (PMC10172293; doi:10.1038/s41431-022-01239-y)
Supplement: Supplementary file 1 — Supplementary Material 1 [file 41431_2022_1239_MOESM1_ESM.docx]

**Supplementary Information S1**

**Quotes from free text responses supporting identified themes**

**Parental choice is facilitated through informed decision making**

*If screening identifies a risk of hearing loss couples can choose to ignore it whilst others can choose to act on it but to remove the choice is unfair. Genetic Counsellor*

**Inclusion of NSHL in a RGCS panel is akin to eugenics and can also express a discriminatory attitude to those living with deafness**

*Developments in modern medicine do throw up ethical challenges - don't want to see a type of eugenics practised in Australia. GP*

*Offering testing for deafness reinforces or provides a perception that deafness is a problem that needs to be eliminated. Genetic Counsellor*

**Defining the severity of deafness is complex**

*Congenital non-syndrome deafness has the potential to be a serious disability with deleterious effects on mental development but if the baby is identified early, fitted with appropriate amplification and commences hearing early intervention by 6 months of age, they can expect age appropriate communication outcomes for school entry. Paediatrician*

*“… the difference in perspectives, support systems and values that people and those around them can have, and how this can influence their experience of living with/caring for someone with hearing loss. Genetic Counsellor*

**There should be limitations on the reproductive decisions available to prospective parents if NSHL is included in RGCS**

*Screening would allow planning for early intervention.  Screening would allow for [preimplantation genetic testing]. It should not be a part of a plan for abortion. Fertility specialist*

*It's not enough to terminate a pregnancy over. Obstetrician*
