# Supplementary material for: Views of healthcare professionals on the inclusion of genes associated with non-syndromic hearing loss in reproductive genetic carrier screening
Source: Eur J Hum Genet. 2023 Feb 9;31(5):548–54. doi: 10.1038/s41431-022-01239-y (PMC10172293; doi:10.1038/s41431-022-01239-y)
Supplement: Supplementary file 2 — Supplementary Material 2 [file 41431_2022_1239_MOESM2_ESM.pdf]

Population wide reproductive carrier screening and deafness questionnaire

Thank you for taking the time to complete this questionnaire which aims to gather perspectives from healthcare practitioners who may have already been involved, or may be involved in the future, with ordering reproductive carrier screening for patients.
As you may be aware, the government has funded a National research project called Mackenzie's Mission which will examine the feasibility of a population based reproductive carrier screening program.

The current RANZCOG recommendation on reproductive carrier screening states:

> **Recommendation No. 3**: Information on carrier screening for other genetic conditions should be offered to all women planning a pregnancy or in the first trimester of pregnancy. Options for carrier screening include screening with a panel for a limited selection of the most frequent conditions (e.g. cystic fibrosis, spinal muscular atrophy and fragile X syndrome) or screening with an expanded panel that contains many disorders (up to hundreds).

Any genetic condition which is inherited in a recessive manner could be included in an expanded panel of genetic conditions. There are several types of **non-syndromic deafness** which are inherited in this way and could be included in an expanded screening panel. If they were to be included in a reproductive carrier screening panel, couples could then find out if they have an increased chance of having a child who is deaf. Couples could use this information to inform their reproductive choices.
We are researchers from the University of NSW and are carrying out a survey of the views of healthcare practitioners toward population wide reproductive carrier screening for inherited forms of deafness. We would be grateful if you can complete this questionnaire so we can find out your thoughts on the inclusion of genes known to cause non-syndromic deafness in reproductive carrier screening. This questionnaire should take no more than **10 minutes** to complete. If you have any queries about this questionnaire, please contact Lucinda Freeman on lucinda.freeman@health.nsw.gov.au.

Survey Questions on Reproductive Carrier Screening: Deafness

A. About You:

1. What State do you live in?

☐ NSW ☐ VIC ☐ WA ☐ SA ☐ QLD ☐ NT ☐ ACT ☒ TAS

2. What is your role as a healthcare provider?

☐ General Practitioner ☐ Genetic Counsellor ☐ Clinical Geneticist ☐ ENT specialist

☐ Obstetrician/gynaecologist ☐ Fertility specialist ☐ Other, please list: ___________________________

3. Number of years in speciality/work area:

☐ 0 - 2 years ☐ 3 - 6 years ☐ 7 - 10 years ☐ 11- 15 years ☐ Over 20 years

4. What is your gender?

☐ Female ☐ Male ☐ Prefer not to say

B. Experiences with reproductive carrier screening

5. Have you offered reproductive carrier screening to patients in the past for any condition?

☐ Yes ☐ No ☐ Unsure

6. Have you offered reproductive carrier screening for the most common genetic conditions as part of a 3-gene panel (cystic fibrosis/spinal muscular atrophy and fragile-X syndrome) ?

☐ Yes ☐ No ☐ Unsure

7. Have you offered reproductive carrier screening for an expanded panel of genetic conditions which can include up to several hundred genetic conditions?

☐ Yes ☐ No ☐ Unsure

8. Do you intend on offering reproductive carrier screening in the future?

☐ Yes ☐ No ☐ Unsure

C. EXPERIENCE WITH PATIENTS WHO HAVE HEARING LOSS

9. How often do you see patients with hearing loss?

☐ Never ☐ Hardly ever (once a year) ☐ Occasionally (Once a month) ☐ Frequently (Every week)

D. VIEWS ON NON-SYNDROMIC DEAFNESS GENES IN REPRODUCTIVE CARRIER SCREENING

10. There are several types of non-syndromic deafness that are inherited in an autosomal recessive manner. Do you think the relevant genes that cause moderate to profound deafness should be included on an extended panel for population wide reproductive carrier screening?

☐ Yes ☐ No ☐ Unsure

11. We are interested in why you think genes for moderate to profound deafness should or should not be included in an extended panel of genes for population wide reproductive carrier screening. The following questions ask you to indicate how much you agree or disagree with a set of statements. Could you please indicate the response that fits best with how you feel about each statement.

For these questions 'deafness' refers to non-syndromic moderate to profound bilateral hearing loss.

	**Strongly Agree**	**Agree**	**Neither agree nor disagree**	**Disagree**	**Strongly disagree**
Moderate to profound deafness is a serious disability.	1	2	3	4	5
Most couples would want to know about their chances of having a moderately to profoundly deaf child.	1	2	3	4	5
Deafness is a disadvantage in a hearing world but is not a disability.	1	2	3	4	5
Couples participating in expanded reproductive carrier screening should be able choose whether to know about their chances of having a deaf child.	1	2	3	4	5
Deafness is not a condition that warrants consideration of reproductive choices.	1	2	3	4	5
There are good treatment and management options for deaf children.	1	2	3	4	5
Being deaf has very little impact on a child's growth or physical development.	1	2	3	4	5
Being deaf has very little impact on a child's mental development.	1	2	3	4	5

12. Do you have any other comments you would like to add about population wide reproductive carrier screening for deafness?

Free text box

Copy of report

If you would like a copy of the report on the study findings, please provide your name and email address. (We will not share your personal contact details or use it in any research; your details will only be used to send the report to you).

☐ Yes, email address: ____

Closing

Thank you for taking the time to complete this questionnaire.
